# College and Hospital Notes

**Published:** 1901-02

**Authors:** 


### COLLEGE AND HOSPITAL NOTES.

At the Buffalo Hospital of the Sisters of Charity the annual meeting and election of officers of the active staff was held on Wednesday, January 9, 1901, with the following result: President, Dr. L. G. Hanley; vice-president, Dr. H. C. Buswell; secretary, Dr. Nelson W. Wilson; executive committee, Drs. J. J. Mooney, Eugene A. Smith, W. D. Greene. Dr. C. M. Daniels was tendered a re-election as secretary, but announced that he could not serve. The staff, however, in recognition of Dr. Daniels's hard work in the interests of the institution unanimously re-elected him in spite of his statement. On his positive declination of the office, a new ballot was ordered and Dr. Wilson was elected. Following the meeting, a banquet was served to the staff by the hospital management.

Dr. Hanley has appointed the following committees: On nurses —Drs. S. Y. Howell, C. M. Daniels, Geo. H. Westinghouse. On hygiene—Drs. De Lancey Rochester, A. J. Colton, Charles S. Jewett. On internes—Drs. H. Y. Grant, B. H. Daggett and Max Kaiser.

Reports made at the annual meeting showed the hospital to be in excellent condition. The wards are well filled and private rooms have seldom been vacant during the year just ended. The next meeting of the staff will be held on the second Wednesday in March.

---

The New York School of Clinical Medicine has opened up a new department of neurology—namely, the study of the neuroses and psychoses of spirit and drug diseases. Dr. T. D. Crothers, of

Hartford, Conn., has been elected professor, and will deliver lectures and give clinical instruction on inebriety from alcohol, opium, cocaine and other narcotics, particularly on the symptomatology, treatment and medico-legal relations. These lectures will begin February 18, 1901, in the lecture room of the college, 328 West 42d Street, New York City. This is the first effort to give special systematic instruction on these diseases, and indicates a great want in the profession of scientific knowledge in this field.

---

OAK GROVE, Flint, Mich., is a hospital for the treatment of nervous and mental diseases and is presided over by Dr. C. B. Burr, a most distinguished alienist and neurologist. The hospital is delightfully located according to any and every viewpoint. It enjoys the shade of stately oaks, the plateau on which its splendid group of buildings stands receives abundance of fresh air, while protected from the severity of high winds, its water supply is pure, and its drainage ample. Adding to these natural conditions, art has done its best in the way of buildings with most modern methods of construction as to plumbing, ventilation, and sunlight, so that the result is a hospital especially adapted to the purposes for which it is intended, which embraces also the comforts and even luxuries of a modern home.

A most artistic calendar for 1901 has lately been issued by the hospital that contains photogravure scenes, which amply illustrate the leading features of this splendid institution.

---

THE medical college of Western Reserve University, Cleveland, O., stands third among the medical colleges of the country in respect to the amount of its endowment. With it are associated the three largest and most complete hospitals in Cleveland, placing at the disposal of its students 700 beds in hospitals as a clinical field, during the greater part of the school year, supplementing the laboratory side of teaching with the practical clinical side in an eminent degree. Therefore students of this college have the very best facilities for a practical medical education.

---

THE Sheppard and Enoch Pratt Hospital, located at Towson, near Baltimore, Md., is a thriving institution for the care and treatment of patients suffering from mental and nervous diseases, presided over by Dr. Edward N. Brush, who is well known in Buffalo. The ninth annual report of the hospital is before us, from which it would appear that since the union of the two benefactions, indicated by its name, the prospects of the institution have become very bright. Especially would this seem to be the case since the hospital is engaged in studies looking toward a better knowledge of insanity, which may be regarded as one of its most important duties. It has been charged that psychiatry has not kept pace with the scientific advances of medicine in general. We do not think the statement is quite true; neverthe-less, this is not the place in which to discuss the question.

What we desire to point out is, that few hospitals of this kind are prepared to afford students opportunities for study and for research work in their laboratories; hence the position which the Sheppard and Enoch Pratt Hospital has taken will prove of great interest to the profession of medicine.

---

THE Buffalo State Hospital has issued its twenty-ninth annual report to the State Commission in Lunacy. Dr. A. W. Hurd, the superin-tendent, is well known as an accomplished alienist and under his administration the hospital is making subtantial progress. The recent addition of the Elmwood building for acute cases has enabled the hos-pital management to deal with these cases in a much more satisfactory manner. It is to be noted that the percentage of recoveries in recoverable cases admitted is 49.71 per cent., while the percentage of recoveries to the total number of admissions is 10.67 per cent. This shows the effect of modern methods in the management of mental and nervous diseases.

---

THE Buffalo Eye and Ear Infirmary presents its twenty-third annual report. From this it would appear that the infirmary is progressing on the lines mapped out in its original charter. The number of new cases treated during the year is 2,905.

---

### Book Reviews.

---

MODERN MEDICINE. By JULIUS L. SALINGER, M. D., Demonstrator of Clinical Medicine, Jefferson Medical College; Hematologist to the Jefferson Medical College Hospital; Pathologist to the Lying-in Charity Hospital, Philadelphia; Assistant Pathologist to the Philadelphia Hospital. And Frederick J. Kalteyer M. D., Assistant Demonstrator of Clinical Medicine, Jefferson Medical Col-lege Hospital; Hematologist to the Jefferson Medical College Hospital. Octavo, pp. 801. Illustrated. Philadelphia and London: W. B. Saunders & Co. 1900. [Price, cloth, $4.00 net.]

It was a great task which Drs. Salinger and Kalteyer set out to perform when they undertook to produce a book on medicine, for at first glance, one would imagine that the field was just about full as

